# Exploring the Social and Stigma-Related Lived Experiences of Pediatric Cancer Survivors in a Canadian Province

**DOI:** 10.3390/curroncol33080494

**Published:** 2026-08-20

**Authors:** Rasel Siddique, Georgia Skardasi, Kayla Crichton, Lisa Goodyear, Teri Stuckless, Holly Etchegary, Sevtap Savas

**Affiliations:** 1 Division of Population Health and Applied Health Sciences, Faculty of Medicine, Memorial University of Newfoundland, St. John’s, NL A1B 3V6, Canada; 2 Division of Biomedical Sciences, Faculty of Medicine, Memorial University of Newfoundland, St. John’s, NL A1B 3V6, Canada; 3 Oncology/Hematology, Janeway Children’s Hospital, St. John’s, NL A1B 3V6, Canada; 4 Discipline of Oncology, Faculty of Medicine, Memorial University of Newfoundland, St. John’s, NL A1B 3V6, Canada

**Keywords:** cancer-related stigma, childhood cancers, lived experience, Newfoundland and Labrador, pediatric cancers, qualitative analysis

## Abstract

**Background**: Worldwide, around 400,000 children are diagnosed with cancer every year. Understanding survivors’ social and stigma-related experiences may help address their needs and improve their outcomes. **Objectives**: To explore the social and stigma-related experiences, coping strategies, and support needs of pediatric cancer survivors in Newfoundland and Labrador, a province of Canada. **Methods**: This is a qualitative, cross-sectional study focusing on retrospective participant experiences. Eligibility criteria included being diagnosed with cancer before the age of 18 and being diagnosed or treated in the province. Extensive recruitment activities were employed. Data collection occurred through semi-structured virtual interviews and completion of a sociodemographic survey. Participant interviews were transcribed verbatim, and themes were identified iteratively through inductive thematic analysis. Descriptive statistics were used to define the participants’ sociodemographic characteristics. **Results**: Seven participants were recruited. Thematic analysis identified five major themes: (i) isolation and being treated differently; (ii) support received, coping mechanisms, and support needs; (iii) resilience and interest to give back; (iv) workplace and disability related experiences; and (v) additional impacts of cancer. Our results showed that participants received substantial social support in various ways but inadequate professional mental health support. School was a significant setting for cancer-related stigmatization. Discrimination in the workplace was rare and was disability-related rather than cancer-related. **Conclusions**: Our results show that there are significant issues to address, such as stigma and isolation experienced by pediatric cancer survivors as well as the need to improve the psychosocial support programs offered to them. Our results also show that the participants had distinct lived experiences compared to adult-onset cancer populations. Overall, the findings presented are expected to inform further studies and healthcare-education policies to help address these issues and improve the experiences of pediatric cancer survivors.

## Simple Summary

In this study, we aimed to understand the social and stigmatization-related experiences of pediatric cancer survivors from Newfoundland and Labrador, a Canadian province. For this purpose, we interviewed seven participants aged 18 or older who previously had a pediatric cancer. Analysis of the participant data showed five common themes related to isolation, support, resilience, workplace and disability-related experiences, and additional impacts of cancer. Schools and peer interactions were a significant source of stigmatization. Overall, better mental health support was deemed necessary. Our results provide insight into local pediatric cancer survivors’ experiences and their needs. Our findings may help with further research and development of support programs regarding pediatric cancers.

## 1. Introduction

Pediatric cancers, also called childhood cancers, are a heterogeneous group of cancers that affect children. Worldwide, around 400,000 children are diagnosed with pediatric cancers annually [1]. According to the Public Health Agency of Canada, the most common types include hematological cancers (leukemia and lymphoma), central nervous system cancers (brain and spinal cord tumors), bone cancers, and soft tissue sarcomas [2].

Scientific and medical advances have contributed substantially to the ability to treat pediatric cancers. As a consequence, the number of pediatric cancer survivors has steadily increased, with the United States showing significant gains in survival rates between 1975 and 2019, and Canada having a similar pace [3,4]. However, global disparities exist. Overall, survival rates have improved considerably in high-income countries compared to low- and middle-income countries [5]. This disparity reflects a lack of access to or availability of diagnostics and treatment [5]. In Canada, more than 80% of the children diagnosed with cancer survive at least five years [2,6].

The rising number of pediatric cancer survivors highlights the importance of ensuring their long-term well-being and quality of life. Cancer diagnosis and treatment disrupts children’s lives and, in some cases, their development. Survivors may face challenges in their social, personal, educational, and work lives. Around two-thirds of adult survivors experience long-term effects from the disease or its treatment [7,8,9,10]. Therefore, understanding survivors’ needs and experiences is essential for improving their outcomes and supporting their quality of life [11].

Cancer-related stigma is one potential survivorship issue that needs further understanding and interventions. Stigma is a societal construct where negative beliefs, misconceptions, and stereotypes about certain groups of people lead to labeling, marginalization, and/or restricted opportunities (discrimination) for those affected [12,13,14]. In the context of cancer, these beliefs or stereotypes often arise from misinformation and lack of understanding, and may be fueled by fear of death (that cancer is associated with) or by visible treatment effects (such as hair loss) [13,14,15]. As a result, survivors may face negative attitudes, discrimination, and isolation, which can negatively affect their mental health [13,14,16]. While most cancer-related stigma studies are focused on adult-onset cancer settings (rather than pediatric settings), young cancer survivors too have reported experiencing stigma, social isolation, and difficulties reintegrating into daily life [13,14,15].

Cancer-related stigma is a poorly understood and under-researched issue in Canada. Building on previous research by our lab [15], this study aims to explore the social and stigma-related experiences, coping strategies, and support needs of pediatric cancer survivors in Newfoundland and Labrador (NL). NL is an Atlantic province of Canada with a low population density (around 550,000 people spread in a relatively large area [17,18]). Most of the NL population lives on the island of Newfoundland. Every year, around 14 new pediatric cancer diagnoses occur in this province [19], representing 1.4% of Canada’s total [4]. Essentially, all patients are treated at the Janeway Children’s Hospital (located in the capital city of St. John’s) [20]. Pediatric cancer survivors’ experiences can help identify potential unique features within the local context, thus informing children’s cancer care and contributing to international literature.

With these goals in mind, using semi-structured interviews and qualitative analysis, we examined: (1) the lived experiences of people diagnosed with pediatric cancers in NL and whether stigmatization and discrimination were experienced by them because of their cancer history; (2) whether such lived experiences affected their well-being; and (3) what type of coping mechanisms or support services they utilized or needed.

## 2. Methods

### 2.1. Context

Within the context of this manuscript, being treated differently and isolated can result from people not being aware of or ignoring the realities of children going through cancer, not knowing how to interact with them, or showing extra care or sympathy to children affected by cancer, in addition to cancer-related stigma or discrimination. Participants were provided with the following definitions of stigma and discrimination during the interview (Supplementary Document S1): Stigma happens when we are negatively or unfairly treated by others because of our history of cancer. It may feel like being side-lined and left out because of cancer. Discrimination happens when we have reduced or diminished opportunities because of our history of cancer. It may feel like you are not given what you normally deserve.

### 2.2. Study Design

This is a patient-oriented, cross-sectional, and qualitative research study.

### 2.3. Eligibility Criteria

Inclusion criteria included being 10 years or older, up to 39 years [21,22], to allow for exploration of current stigma-related experiences (e.g., from the 10–18 year old individuals), and a retrospective look back from the older individuals (19–39 years old individuals); having being diagnosed with pediatric cancers before the age of 18; being able to provide consent/assent (with consent by a legal guardian when the participant is a minor); and having being diagnosed with and/or treated for pediatric cancers in NL.

### 2.4. Recruitment

Recruitment via convenience sampling occurred between November 2024 and November 2025. Various recruitment methods, venues, and contacts were utilized to recruit participants. These included healthcare providers in the Pediatric Oncology program at the Janeway Children’s Hospital; patients’ circle of care and other settings where aftercare is provided (e.g., Dr. H. Bliss Murphy Cancer Centre); relevant cancer organizations (e.g., Candlelighters NL [23], Young Adult Cancer Canada [24]); social media (e.g., Twitter posts, LinkedIn posts, paid Facebook advertisements); Memorial University listserv circulations; word of mouth; posters displayed in different parts of the University, community spaces, the provincial Cancer Care clinic and Janeway Hospital; NLSUPPORT [25] communications; and main stream media articles (e.g., [26]), interviews, and Public Service Announcements (VOCM news station [27]). We also posted information about the study on our website [28].

Three collaborators were in patients’ circle of care (Dr. Teri Stuckless, Dr. Gerard Farrell, and Dr. Lisa Goodyear), who distributed the study information letter to eligible patients whom they see in the clinic.

Additionally, in September 2025, we mailed out the study information letters to eligible individuals diagnosed with pediatric cancers in the province and recorded within the provincial cancer care registry (*n* = 390).

### 2.5. Potential Fraudulent Participation Control Measures

On 3 February 2025, the PI posted study information as a paid advertisement on Facebook (which was also posted on other social media accounts, such as LinkedIn, Twitter, and Bluesky), which resulted in receiving spam and more than 100 emails, with most if not all coming from Gmail accounts. This, combined with the rarity of pediatric cancers, raised serious concerns about the possibility of fraudulent study participation attempt by individuals who would like to take advantage of the study compensation. These concerns led to changes in our consent and eligibility determination processes.

Working with the Health Research Ethics Authority (HREA) office and ethics officer, and reaching out to other researchers, a series of measures were implemented and approved by the Health Research Ethics Board (HREB). These measures included: (1) removing compensation information from posters and social media posts; (2) implementing a pre-screening session with potential participants (via phone, virtually, or in person) prior to consenting; and (3) verifying the personal identity used by the potential participants when contacting the research team.

During the pre-screening sessions, potential participants were asked specific questions from the sociodemographic survey (e.g., their age, type of cancer they were diagnosed with, their treatment center, age of diagnosis, childhood town), and their responses were recorded. If they satisfied the eligibility criteria and consented to participating in the study, the same questions were asked again (via an email prior to the interview, or at the beginning of the interview before starting the recording) to assess the consistency of their responses over time. If inconsistent responses were detected at this stage, the process was ended by thanking participants and informing them that their responses did not match the information previously provided or that they were not eligible to participate in the study.

To confirm that the names used by the potential participants while contacting the research team matched their personal identity, we required the video to be on during the interview process to help identify potentially duplicate participants, and required confirmation of their identity (prior to start of the interview recording) by presenting a picture ID (e.g., school ID, extra-curricular activity ID, work ID, driver license, provincial resident ID, passport) or an item mailed to their name and address. This documentation was not recorded.

### 2.6. Consent Process

Interested participants were advised to contact the study PI (SS). As explained above, a prescreening session was held with potential participants to confirm their eligibility via phone or WebEx, the approved virtual platform of Memorial University [29]. Eligible individuals were sent the consent form and sociodemographic questionnaire via email. If no response was received, individuals were reminded after a week, and consent was obtained via phone or email. While all participants in this study were 18–39 years old, for participants younger than 18 years of age, we had planned to use the parental/guardian permission/consent form and assent form.

### 2.7. Participant Compensation

Each consented individual was offered a digital gift card in the amount of CAD25.

### 2.8. Collection of Sociodemographic Data

We emailed a Word copy of a survey to collect the sociodemographic information of the consented participants. Participants had the flexibility to complete it via phone, virtually via a WebEx [29] session, or via email.

### 2.9. Interviews

Interviews were conducted virtually using WebEx [29], enabling us to interview the respondents from remote and rural areas of the province, thereby increasing the equity and inclusiveness of this study. To help with the interview process, separate checklists were prepared for adult and minor participants. The graduate student (RS) was trained to conduct the interview via several mock interview sessions. The interviews were semi-structured and focused on the social and stigma-related experiences of pediatric cancer patients/survivors, their support needs, and the coping mechanisms that they utilized or needed. Interview questions were informed by literature review, lived and clinical experiences of team members, and prior work of the team [12,15,30,31,32,33] and focused on the following themes: (1) lived experiences and any issues experienced following their cancer diagnosis (in social, school, healthcare, and if relevant, work environments); (2) support they have received from others following their cancer diagnosis (in social, school, healthcare, and if relevant, work environments); (3) lived experiences regarding specifically stigma or discrimination because of cancer following their diagnosis (in social, school, healthcare, and if relevant, work environments); (4) effects of stigma and discrimination on their well-being; (5) social support utilized and types of coping mechanisms applied to address cancer-related stigma and its consequences; (6) whether clinical support was sought or received in order to cope with stigma’s effects. The interview guide for adult participants is shown in Supplementary Document S1. Interviews lasted on average around 38 min (range: 25–58 min). The interviews were conducted by the graduate student (RS), and his supervisor (SS) attended the interview sessions as an observer with their camera off. The interviews were recorded by WebEx and transcribed verbatim [34]. Transcripts were verified by two assistants (GS, KC) along with RS, and disagreements were resolved by the supervisor (SS) prior to thematic analysis.

### 2.10. Data Analysis

Descriptive statistics (i.e., frequency tables) were used to describe the sociodemographic data collected from the study participants (Supplementary Document S2). The themes in participants’ interview data were identified by qualitative analysis [34,35]. We used the inductive thematic analysis approach where themes arise from the interview data [34,35]. Verbatim transcripts were coded iteratively by RS in an inductive manner [34,35]. These initial codes were then assessed by a second analyst (SS) who reviewed all transcripts for coding accuracy. This step was followed by discussions between the two analysts to finalize the codes. The relationships among the codes and themes extracted were examined carefully and summarized in figures by RS, and then reviewed, discussed, and finalized by RS and SS. Since all participants were 18 years or older (and no participant younger than 18 years of age was recruited), this study should be considered as a retrospective look at the adult survivors’ experiences.

## 3. Results

Participant characteristics are shown in Supplementary Document S2. In brief, all seven participants were White/had European ancestry, and most had worked after their diagnosis and were living in an urban area. Four of the participants identified as men and three identified as women. Participants represented various age ranges, with a median time since diagnosis of 20 years. The youngest participant was 18 years old.

Thematic analysis of the interview data generated five themes: (i) isolation and being treated differently; (ii) support received, coping mechanisms, and support needs; (iii) resilience and interest to give back; (iv) workplace and disability-related experiences; and (v) additional impacts of cancer.

### 3.1. Social Isolation and Being Treated Differently

**Figure 1:**
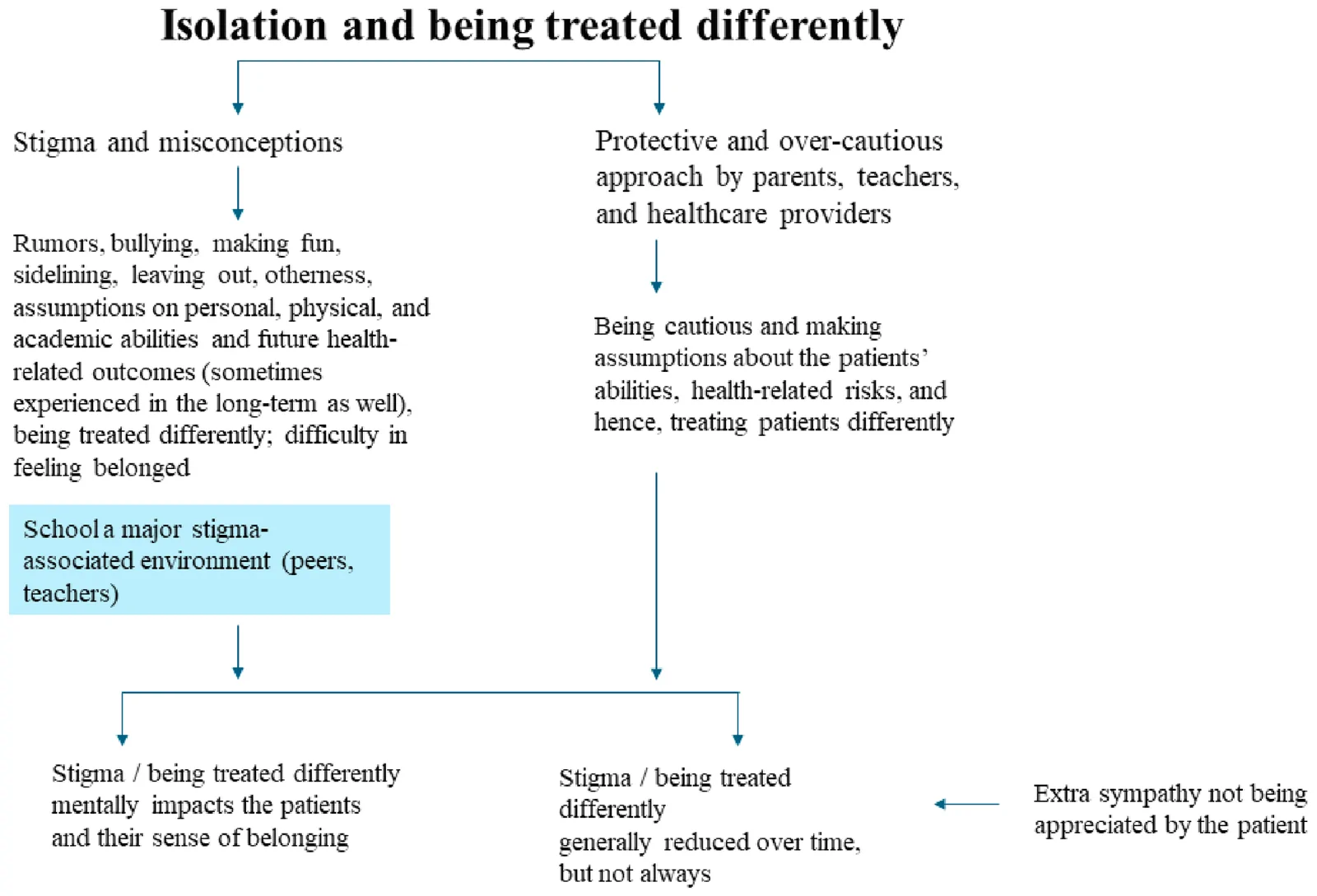
Theme: Isolation and being treated differently.

After their cancer diagnosis, all participants were treated differently in a positive or negative way (Figure 1; Table 1). Except for one, all participants reported experiences of stigma, labeling, isolation, otherness, and assumptions made about them.

Results showed that social relationships were affected. Some participants experienced restricted social life, altered relationships with family members, and experienced nosiness, curiosity, and insensitive questions from others about their disease, leading to discomfort. In some cases, the latter experiences happened long after their cancer diagnosis.

Interestingly, stigma, assumptions, and misconceptions related to cancer and the protective approach by those around the participant contributed to their isolation and being treated differently. Specifically, in two cases, participants reported being isolated from others for protective-preventive reasons (e.g., by protective parents, friends, or doctors aiming to limit potential environmental exposures and physical harms). On the other hand, participants shared experiences where people from their environment, such as friends, parents, teachers, and doctors, also made assumptions about or neglected patients’ personal and academic abilities, symptoms, and future health outcomes (such as fertility). Four of the participants were called “cancer kid” by members of the community, which sometimes stuck with them for a long time. Negative assumptions by healthcare providers were rarely experienced and related to fear of cancer in adult years (e.g., one participant was called a “dramatic patient” as an adult) but nonetheless led to frustration.

Misconceptions about cancer (such as cancer being contagious, leading to isolation by peers or their parents) or being made fun of and pitied were reported by four participants, suggesting stigma was a common experience during childhood years. Teachers were mainly supportive, considerate, and protective. However, school emerged as a major environment for stigma and isolation. Some participants reported changes in teachers’ attitudes, a lack of understanding of student’s physical limitations/pushing them beyond their limits, isolation from peers (e.g., having lunches alone), experiences of otherness, exclusion from activities, and peer bullying—these sometimes caused adverse effects on their mental health. In one case, a participant experienced extra sympathy at school, where they were treated favorably because of their cancer diagnosis.

Consequences of isolation, assumptions, and stigma on participant well-being varied. Three participants said that stigma or discrimination due to cancer negatively affected their well-being. For example, one participant had struggled with social exclusion and isolation from friends and bullying at school, which impacted their psychological and mental health. Another participant recounted a similar experience in their social interactions. Developing or retaining friendships was difficult for one participant and mentally affected them severely. One participant explicitly stated that they needed external support to address the effects of cancer-related stigma on their well-being, but they did not utilize it—it is not clear whether this was a barrier-to-access issue.

Potential self-stigma was identified but was not common. One participant blamed themselves for what had been happening in their life. Another participant occasionally felt different because of the academic challenges they faced and their inability to play contact sports as before, hinting at self-stigma.

Overall, often times early experiences of stigma, isolation, and being treated differently reduced over time. Experiencing stigma hardly continued into older age, but for two participants, being treated differently continued over time.

Interestingly, discrimination was scarce and linked only to disability (see below). Also, participants did not report discrimination in romantic relationships or career opportunities due to cancer.

### 3.2. Support Received, Coping Mechanisms, and Support Needs

**Figure 2:**
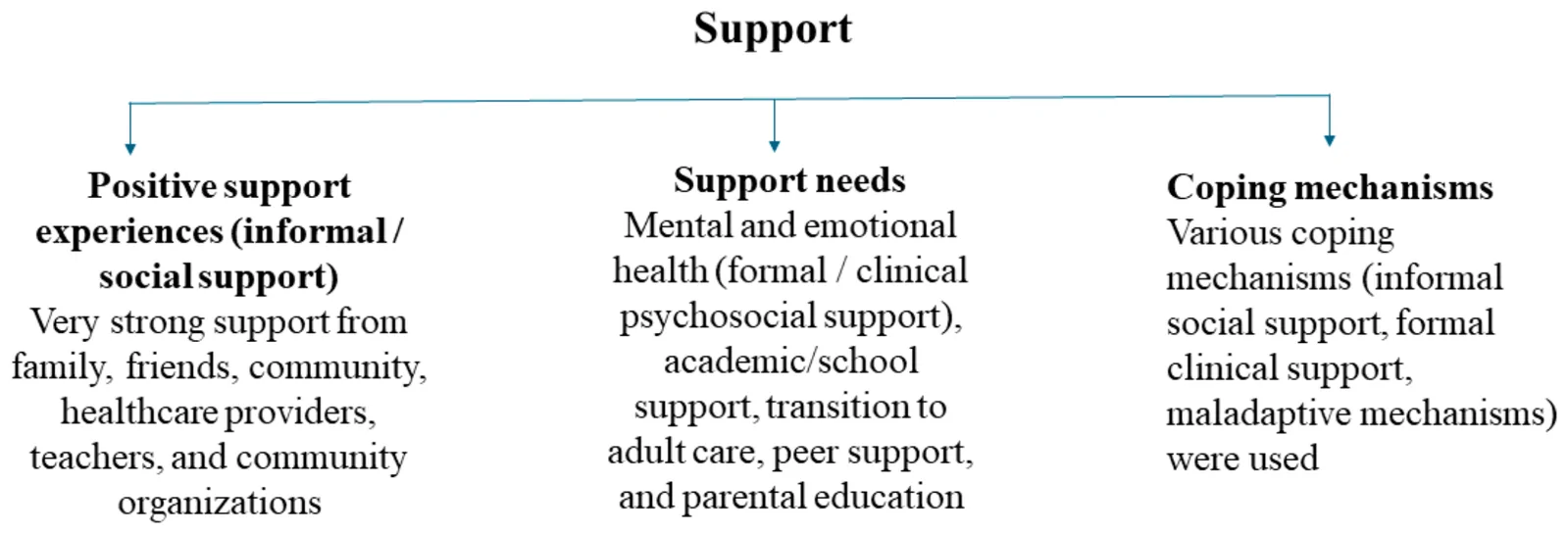
Theme: Support.

Participants expressed that following their cancer diagnosis, they received strong and various types of non-clinical support (e.g., financial, spiritual, social and emotional support, charity organizations’ programs providing affordable out-of-home accommodations, workplace accommodations provided to a parent, and encouragement to overcome obstacles) from different sources, including family, friends, healthcare providers, teachers, peers, community support organizations (NL Candlelighters [23]; Children’s Wish/Make-a-wish Foundation [36], Ronald McDonald House [37], Janeway Hospital [20]), community, and churches/church goers (Figure 2; Table 1).

Overall, support and sympathy were valued, but four participants stated that additional support, especially in terms of schooling and peer support, would be desired. As expected, the support levels had generally decreased over time (expressed by four participants). Also, one participant emphasized the importance of proper transition from pediatric to adult care, a well-known care need in pediatric cancers.

The need for, and appreciation of, proper psychosocial support was evident. A variety of mechanisms were utilized by participants to cope with cancer-related stigma, isolation, or cancer as a whole (including formal and informal psychosocial support and maladaptive practices). These included social networking with others affected by pediatric cancers and participating in the Children’s Wish/Make-a-wish [36] and Candlelighters NL/Camp Delight programs [23]; talking to friends, family, and healthcare providers; public speaking/writing and advocacy; determination to feel normal, having a positive attitude; utilizing support groups, medications, counseling; letting go of others’ opinions; and engaging in faith-based activities-prayer, as well as maladaptive coping mechanisms such as avoiding conflicts; watching TV; spending time in bed; forgetting the past; dissociation; and playing video games. However, there was also a strong need expressed for better coping strategies and resources (e.g., via proper mental health care; local support groups; educating the parents regarding the importance of mental health support).

### 3.3. Resilience and Interest to Give Back

**Figure 3:**
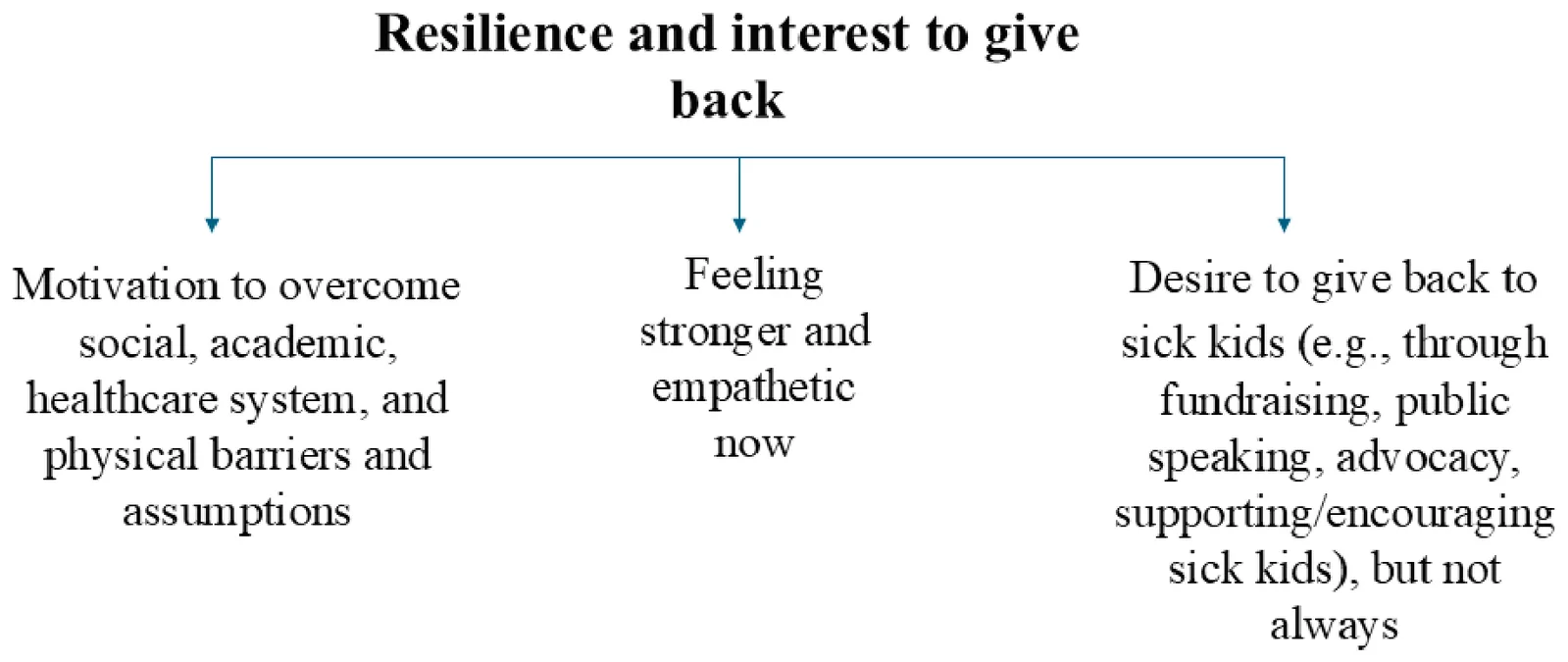
Theme: Resilience and interest to give back.

While not all, some of the participants were very interested in giving back to the community (e.g., via advocacy, fundraising activities, public speaking, connections with kids who were diagnosed with cancer). In addition, a particularly strong sense of determination and resilience was evident in two of the participants, who focused on overcoming assumptions/obstacles, had a positive attitude, desired to move on, and converted experiences with cancer into becoming stronger or more empathetic (Figure 3; Table 1).

### 3.4. Workplace and Disability-Related Experiences

**Figure 4:**
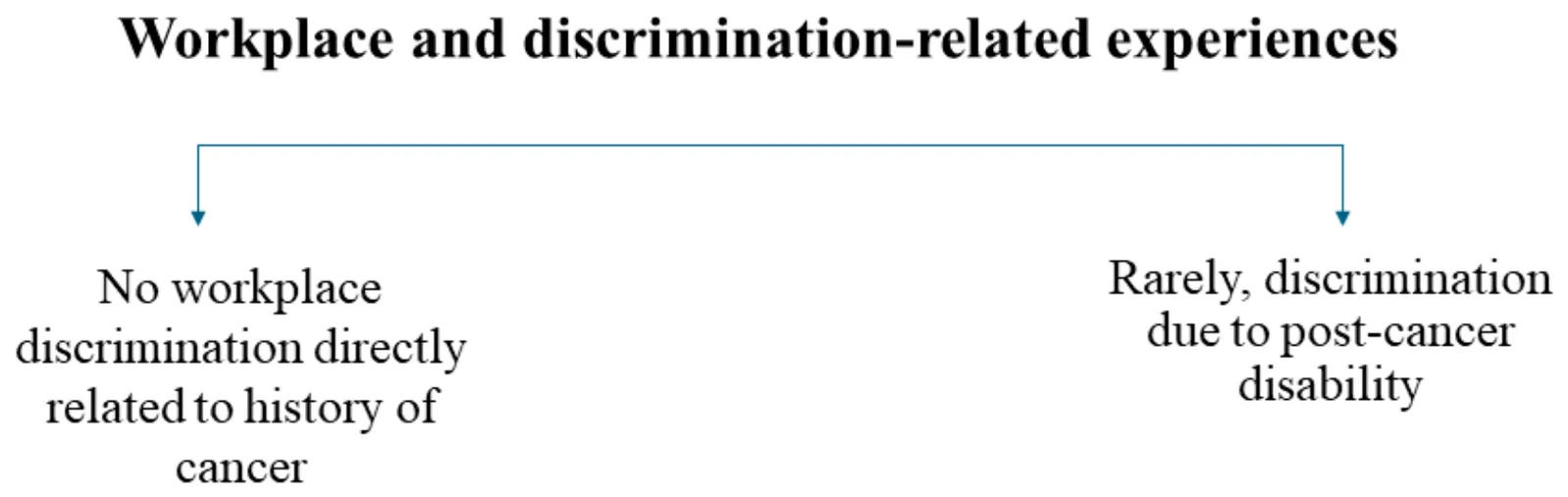
Theme: Workplace and discrimination-related experiences.

Participants did not report workplace difficulties directly due to cancer history. However, two participants experienced workplace issues, one being discrimination, but it was due to disability, rather than cancer (Figure 4; Table 1).

### 3.5. Additional Impacts of Cancer

**Figure 5:**
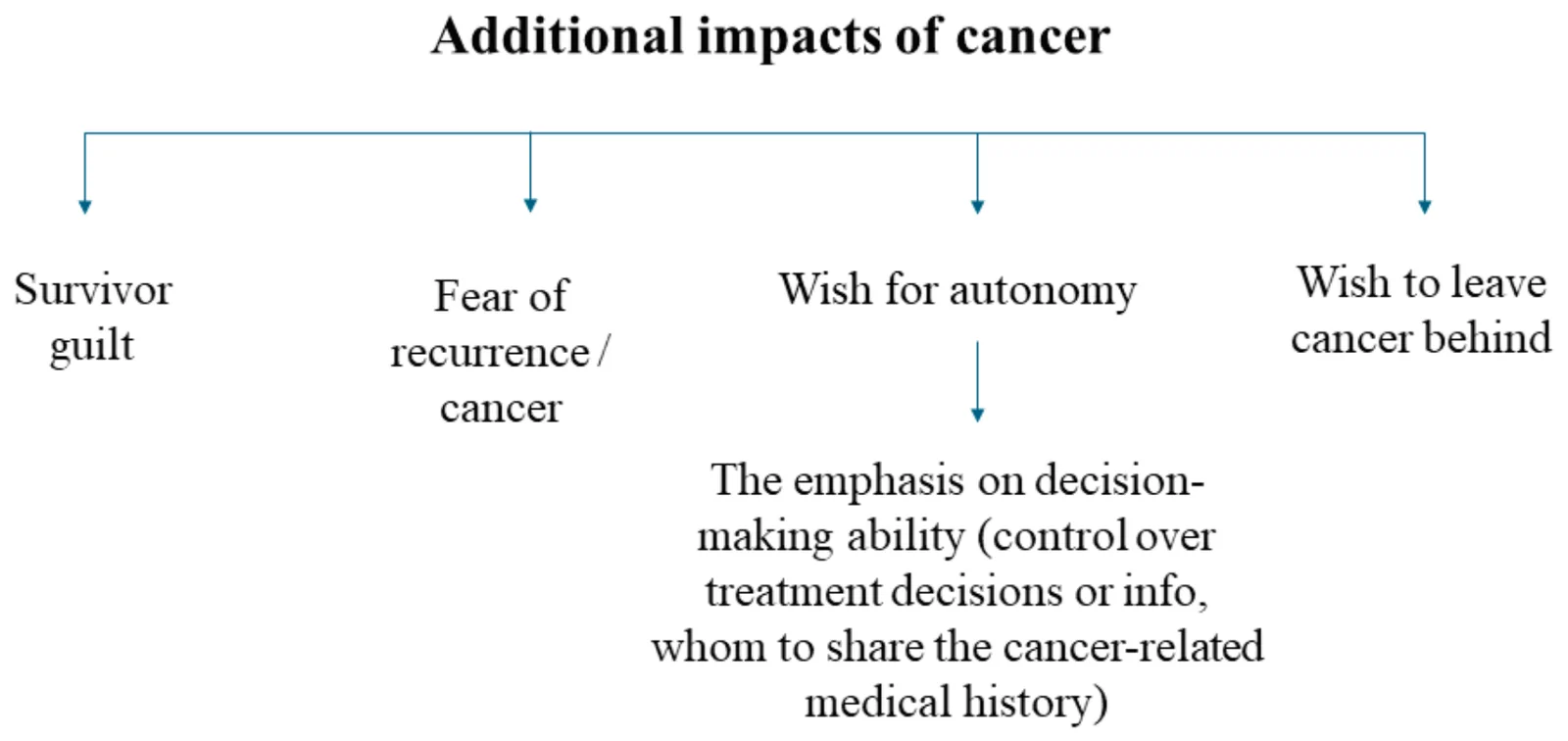
Theme: Additional impacts of cancer.

While some of the participants did not mind disclosing their cancer history to others, it was not a practice adopted by everyone. Two participants reacted to their cancer history being disclosed without their consent. One participant expressed not being able to have autonomy regarding decision-making and having access to their medical information kept by parents. The wish to leave “cancer” behind was also apparent (*n* = 3 participants). Two participants expressed fear of recurrence or having a new cancer diagnosis being a part of their adult lives. Last, survivor guilt was experienced by most (*n* = 5) participants, ignited by inspirational words such as “*You are a hero*”, uttered by family, friends, and peers. In most cases, this kind of talk made the participants uncomfortable (Figure 5; Table 1).

## 4. Discussion

This study explored lived experiences of pediatric cancer survivors in NL. Analysis of the participant data identified key themes and provided insight into aspects of their well-being, their social and academic lives, and experiences of pediatric cancer-related stigma. Our results contribute to existing literature on these topics, provide preliminary evidence to help improve local policies, healthcare and support systems for children affected by cancer, and create opportunities for further research, advocacy, education, and public discourse that can help address isolation, misconceptions, and stigma associated with pediatric cancers.

Participant narratives emphasized the importance of formal/clinical psychosocial support services and their accessibility by children affected by cancer and adult survivors. In the present study, both positive and negative impacts and social outcomes experienced by participants were delineated. While participants recalled substantial and heterogenous support following their cancer diagnosis, three of them noted a significant need for—and a lack of—adequate clinical mental health support (e.g., insufficient access to clinical psychosocial care) that may be essential for quality of life [38,39]. As a result, a considerable portion of coping mechanisms utilized were “escape” mechanisms (e.g., spending time in bed; dissociation). Also, initial support was generally reduced over time, leaving long-term support needs sometimes unaddressed.

Specific areas that can benefit from formal psychosocial support, sometimes during adulthood as well, were experiencing stigma and isolation as well as fear of recurrence/cancer and survivor guilt. Pediatric cancer survivors are at a higher risk of subsequent malignant neoplasm [40] as well as experiencing fear of recurrence (or cancer), a well-known and distressing experience [41,42,43,44]. Considering its impact on the mental well-being of survivors, detecting [45] and addressing this fear by interventions may prove useful [42]. Another area of attention identified was survivor guilt [46]. In our study, survivor guilt had arisen due to loss of peers affected by cancer and was expressed by most (five out of seven) participants. Current and future psychosocial support services in pediatric cancers may want to integrate survivor guilt and associated self-blame among the psychosocial symptoms to address [47].

Six out of seven participants described experiencing stigma, labeling, isolation, otherness, or having assumptions made about them. While cancer-related stigma has been widely studied in adult-onset cancer settings, very little is known about pediatric cancer-related stigma. Within the context of cancer, stigma can be understood as an invisible yet strong barrier that limits survivors’ ability to fully reintegrate into society despite medical recovery. Stigma can be categorized as self-stigma (where negative stereotypes and prejudice are accepted/internalized by the person), public (also called social stigma, where stigma can be enacted by others during interactions), and structural (also called systemic stigma), where policies and practices lead to stigmatization of certain groups of people [48]. Stigma can also be enacted (i.e., experienced) or perceived-anticipated [49]. In our study, most stigma identified was social stigma enacted by others. In contrast to stigma, discrimination (reduced or diminished opportunities) was rarely expressed by the participants, and when it occurred, was related to post-cancer disability rather than their cancer history.

In this study, peer interactions and school environment were identified as key sources of stigmatization, as five participants experienced isolation and/or bullying by peers and differential treatment by peers and teachers. Physical disfiguration—such as hair loss—as well as extra sympathy or care due to disease or treatment were major drivers of these stigmatizing experiences. Peer bullying of children with cancer in school environments is identified in other studies [50], which is concerning and can be mitigated. Proactive measures, such as educational programs, may reduce stigmatization at schools. In addition, psychosocial and peer support for children affected by cancer may be offered to address the impact of stigma on them [48].

Isolation and loneliness were common experiences in social and school environments of participants. Interestingly, we found that stigma-related isolation and/or protective isolation were experienced by all participants. Isolation is a common experience in cancer survivors that can lead to distress and negative experiences [51,52,53,54]. The development of support programs addressing social isolation among young survivors can benefit this particularly vulnerable demographic group in NL. Understanding the impact of protective isolation and, where needed, mitigating the risks associated with it, should also be one of the priorities, so that children can access healthier opportunities to overcome the negative consequences of isolation due to cancer.

Despite experiencing serious challenges, such as stigmatization and isolation, select participants also showed resilience (*n* = 2), a desire to move on (*n* = 3), and a desire to give back to the community through helping others and advocacy (*n* = 2), suggesting that they adjusted to adulthood and/or managed the impact of cancer to some extent. Resilience has similarly been identified in other studies focusing on children with cancer [55,56]. While we did not examine the factors leading to resilience, it is possible that the myriad of social, familial, and community supports may have made them optimistic and determined. Further studies on resilience may help develop survivorship strategies and guidelines in pediatric cancer settings.

Pediatric cancers differ from adult cancers in several aspects, including in terms of their rarity, types, treatment, causes, survival rates, and symptoms that are often unknown or vague [57]. These distinctions have important implications for pediatric cancer diagnosis and treatment. There are also differences in the social and stigma-related experiences between adult-onset and pediatric cancer survivors. For example, in this study, participants did not report any workplace issues due to cancer history. In contrast, studies in adult-onset groups showed that patients experienced discrimination and challenges, particularly at the workplace [15,16,58]. In a recently published qualitative study, our group examined the social and workplace experiences of adult-onset cancer survivors in the same Canadian province [16]. When these results and the study results are compared, it is apparent that there are shared experiences between these two demographic groups, such as peer support and emotional support needs. However, compared to the study population, adult-onset cancer survivors had different issues, such as financial toxicity, insurance-related issues, workplace discrimination, accommodation needs, and information needs. It is interesting that many adults reported a loss of income related to their diagnosis; however, one participant here indicated their father’s workplace authority assured that they “*wouldn’t miss a paycheck*” while the family was going through treatment (Table 1). In addition, although stigma also exists in the adult-onset cancer settings, it sometimes manifests in complex ways (e.g., genderized stigma). These comparisons show the different needs and experiences of pediatric and adult-onset cancer survivors, even within the same province. Factors contributing to these differences may be the age at diagnosis (necessitating parents shouldering economic- and insurance-related burdens) and young peers witnessing the sickness and treatment effects in social and school environments (rather than the workplaces in adult settings), leading to unintentional stigma, being treated differently, or the exclusion of pediatric cancer survivors. In addition, adults diagnosed with cancer may have a level of stigma attached as their cancers may be perceived to be because of their actions (such as smoking), which may not be the case for children. This may lead to lower levels of stigma or discrimination in pediatric cancer patients—this area needs further research.

Lastly, many of the issues expressed by the participants are universal. The Global Initiative for Childhood Cancer by the World Health Organization aims to improve survival and outcomes for the global pediatric cancer population [59,60]. In Canada, there have been recent concerted efforts, such as the national ACCESS network [61] that aim to understand the disease biology and improve the well-being and treatment of pediatric cancer patients. These large-scale initiatives and independent research programs are promising and expected to help address the support needed and other issues experienced by pediatric cancer patients.

Strengths and limitations: This study used large numbers of recruitment strategies over a year and recruited seven participants. Despite the small number of participants, data were rich and allowed us to identify key themes. However, we cannot rule out the fact that more themes could be discovered if more participants were included. The study included participants who self-identified as White/having European ancestry and who were mostly from urban areas; hence, it did not have enough diversity among the participants. Our study also included participants with various types of cancers; hence, it did not produce cancer-site-specific data on the social and other experiences of survivors. In this study, there were no participants younger than 18 years of age, which limited the evaluation of the lived experiences of pediatric cancer patients recently diagnosed. Therefore, this study presented retrospective accounts of adult pediatric cancer survivors. However, having adult survivors as participants helped identify long-term cancer and stigma-related lived experiences. Possible bias introduced by convenience sampling and recall bias should be kept in mind while interpreting the data. We believe that the prescreening procedures significantly reduced the risk of fraudulent participation, while also potentially discouraging eligible participants. The use of the virtual platform helped include participants from across the province. The interviewer (RS) was trained prior to interviews and closely followed the interview checklist and guideline document to reduce the variability among different interviews. Transcripts were reviewed, corrected, and finalized by four team members, increasing the accuracy of the participant data. Careful data examinations were performed by two analysts to increase the reliability of the coded data and themes. As—to our knowledge—this is the first study examining stigma in pediatric cancers in Canada, our findings also provide important insights on this topic in this country and contribute to the international literature.

## 5. Conclusions

Pediatric cancer survivors may face challenges, including stigmatization, which can negatively impact their well-being. In this study, we explored the lived experiences of seven participants from a Canadian province. Overall, our findings showed substantial social support provided to participants while also pointing out the need for better clinical psychosocial support, the existence of stigma, school environment as the major setting for stigmatization, long-term effects of cancer, and survivor resilience. Our findings help understand the local context and may inform future research, clinical practice, support programs, and policy development to reduce the burden associated with cancer and stigma.

## Figures and Tables

**Table 1:** Example quotes for major themes and subthemes identified in this study.

Themes	Subthemes	Example Quotes (Participant ID)
Isolation and being treated differently	Stigma and misconceptions	“Mostly lonely and kinda wishing I could fit in with the crowd a little bit more.” (A12) “When I was a kid, I was like known as like the cancer kid, like everyone in my town knew that like I was the one.” (A12) “… especially when I was moving to new schools a lot, and I did sometimes feel like I […] never had like the most friends immediately and I felt like I, you know, I was a bit othered so […] make me feel a little lonely.” (A12) “Person that didn’t know me that would like just see my […] and like poke fun at me, or treat me differently because of that.” (A14) “But I think a lot of parents kept kids from me honestly in case I didn’t survive, I did have a couple of friends that kind of distanced and I just didn’t see a whole lot and we didn’t rekindle our friendship after my treatments or anything.” (A15) “As a child, I just always felt like I did something wrong. I didn’t understand it enough.” (A15) “I think in the cases of parents that wouldn’t let their kids hang out with me because they were afraid that their kids were gonna get sick perhaps that way. I had teachers that either felt bad for me because I was sick and miss time and then on the same, on the same hand I had teachers that felt like I shouldn’t be passing a grade because I wasn’t there.” (A15) “I was definitely bullied for, not necessarily for having cancer, but for being bald, for losing all my hair. I was bullied quite a bit. […] there were times yes, that I was definitely treated unfairly because I was sick and that was out of my control, but kids are cruel.” (A15) “I had a […] teacher that would force me to do everything in a […] class, but forced me to go [*a type of sport*] and forced me to do these things. And the whole time I’d be “No, NO, I can’t do that. I’m not allowed to do that. I’m not physically able to do that. And [they] just never took it seriously. [They] didn’t believe a word I said. I had doctor’s notes, I had all the proof that [they] needed, and [they] went out of [their] way to make my life miserable for the things that I couldn’t do.” (A15) “I was an all star [*a contact sport*] player before I got diagnosed […]. After my surgery and my treatment and all that you know I couldn’t play [*a contact sport*] at the same level that I could and so you know what that’s always been, like a sore point for me.” (A16) “In school, I wasn’t able to place, play with like the sports, things like that, I wasn’t able to participate in a lot of school activities. I just had to watch, so I felt very like left out from that aspect, which also like led to less friendships and things like that, so that was through my whole schooling.” (A20) “Yeah, so I definitely had to work hard, harder than I should have had to, to feel included, and to get into and do all of the things that I do cause I like because of that exclusion and lack of friends and bullying I didn’t have the social supports, so I had to work a lot harder.” (A20) “Well, I was always known as the kid who had cancer, so definitely there was some stigmatization there.” (A20) “I was excluded from like partaking in like sports and all that so I was kinda made to like sit out and just watch gym class, which was not really inclusive.” (A20) “In terms of school, it was different. I felt a little isolated just because, like I had to take like different transportation to school. I wasn’t taking the bus with everyone. If I had to go eat lunch, I had to eat lunch on my own.” (A25)
	Protective and over-cautious approach by others	“Teachers always ratted on me if I was doing, if I was doing something I shouldn’t have been or, like playing a sport that I shouldn’t have been. They’d always just call my mom.” (A15) “Especially when I was a kid, it was like a lot of the time I would be left out of, activities because I was seen as like too sickly, like I remember there was a time where I wasn’t allowed to feel like [*a kind of contact sport*] cause a teacher wouldn’t let me because I thought I might get hurt or I had to be left out of like […] ball games or something.” (A25) “I was treated I think a little bit differently just because I think people were more cautious around me health wise and like injury wise and just in terms of physical health and safety stuff.” (A26)
	Extra sympathy not being appreciated by participant	“I had this one teacher that I didn’t really think liked me that much, but after [they] found out I had had cancer and found out about my story and stuff, [they] treated me different and that kind of left a bit of a sour taste in my mouth.” (A14)
Support	Positive support experiences	“One big thing that was really a big support for me was the, the Candlelighters like foundation and they have a summer camp for kids with cancer and stuff, camp delight. And I went there from my 1st year all the way up to age 17, and that’s one of the biggest like support systems I’ve had, I think.” (A12) “My dad’s employer was amazing. He told my dad […] your [child] is sick, take care of your [child]. He said you will not miss a paycheck.” (A14) “So like there was a lot of support and we had a lot of people in our corner looking out for us and also like the Ronald McDonald house, when we made it to […].” (A14) “We did a lot of fundraisers, so we had a lot of financial supports and then, yeah, financial support was big.” (A15) “They were with me pretty much all the time. I was barely left alone in the hospital room […] and I was very close with my […] siblings as well and even after even today it’s same thing, my family is very close and we’re very supportive of each other.” (A16) “My doctors, my nurses were all awesome I would say. They were really helpful, very supportive. I feel like they always did their best when it came to trying to help me. Same thing with teachers.” (A25) “Healthcare providers I think were good with social support, don’t have anything bad to say about them. Everyone was good, friendly, understanding. Teachers and stuff, for the most part I think were good. I don’t have any specific bad memories there.” (A26)
	Coping mechanisms	“I talked a lot to my family and we were, we’re very close family, so I think, you know, that conversation even still occurs sometimes. But, I talked to a lot to my family and to my friends.” (A14) “My sister, she’s great. I do talk to her about it. I do have a, I do have one friend that we talk about it pretty frequently, we did, we don’t so much now but we did, healthcare professionals not really well like once I was into therapy, it wasn’t even healthcare professionals, it was honestly just counselors and therapists.” (A15) “I wasn’t even allowed to exercise, so I didn’t do anything for wellness, really I watched a lot of TV and spent a lot of time in bed.” (A20) “I think I coped mostly, if things were really bad it was sort of, I think I’d try to disassociate. So, I got really into like video games for example, were a big like escape mechanism for me.” (A25)
	Support needs	“Until I was discharged from the Janeway, I had plenty of support. And then once my Janeway discharge, I have nothing. I don’t have an oncologist anymore, I don’t have a team anymore.” (A15) “Maybe a kid’s support group because talking to your parents at […] years old, they just want you to survive. They don’t really care how you’re feeling, not mentally anyway. They just want you to survive.” (A15) “My parents don’t believe in mental health.” (A15) “I’ve never ever spoke to a psychologist of how any of it affected me. So that would have been nice to have been included in my care.” (A20) “The school, again, I didn’t find super supportive, just because I missed a lot of school. I wasn’t offered school while I was in hospital, I was hospitalized a lot, and I wasn’t offered any school supports while I was there, and I wasn’t given anything from the school to kind of catch me up.” (A20)
Resilience and interest to give back	Motivation to overcome barriers and assumptions	“I think it’s made me, like it’s made me strong that like I’m, like again determined to get through stuff.” (A14) “There was a lot of things that doctors thought that I wouldn’t be able to do post cancer that my family and my friends encouraged me to not, you know, just give up on those things and to push through and to try harder and stuff like that, and I overcame all of them.” (A14) “I’d just, you know, did my best to be normal and well normal, trying to, you know, just, yeah, just keep on, just keep on going, that’s all. Right? I wouldn’t, you know, it never held me back in any way, and, you know, I wouldn’t let it hold me back in any way.” (A16) “I’ve always been a go getter/go get any opportunity that I want. My diagnosis has never stopped any of that.” (A20)
	Feeling stronger and empathetic	“Probably made me a stronger person and, able to handle a lot more because, you know, I’ve already handled almost the hardest thing to do when I was […] years old, right? So, yeah, it made me a lot stronger and able to handle a lot more situations.” (A16)
	Giving back to sick kids	“Every couple of years now we’ve been raising money for […].” (A14) “I’ve been able to like share my story in so many different places in so many different ways. Like I’ve told my story on TV multiple times I’ve told my story in front of like stadiums full of people […]. It makes you feel really good that you’re able to provide validation to other people who are going through things.” (A14)
Workplace experiences	No issues due to history of cancer	“Work wise, again, it was post, it was a long time after, so it didn’t affect.” (A14) “Of course […] there’s things that I can’t do, but in the job that I’m in right now there’s not. I’ve never really had an issue at work.” (A15) “In terms of work, it was, if I did ever have anything, cancer related sort of come up, they were super accommodating with like giving me time off and stuff to deal with that so yeah.” (A25)
	Issues due to disability	“So I am disabled […]. Like my, all of my employers have been relatively good. Never had no, not too much of an issue at work besides my disability, but not an issue because it was cancer.” (A15)
	Discrimination due to post-cancer disability that is sometimes caused by cancer	“I have not experienced any, anything cancer related […], any different treatments to cancer wise in the workplace. However, with because of my memory disability I have dealt with some discrimination at work.” (A26)
Additional impacts of cancer	Survivor guilt	“Yeah, people do tend to talk about like that side of things like I’m a super person like I’m super strong, all that stuff and I’m like, No, I was a kid, I did what I had to, to survive. I’m no superhero I’m the same as everyone else that’s definitely very prevalent. I still hear it my family especially, I’m their little survivor, their warrior, all that stuff, but mostly the family.” (A20) “I think that, that pressure sort of gets put on a lot of kids when they survive and then that can be a hard feeling to deal with because you go through cancer with other people and then there’s nothing that you do differently than them, but sometimes they might pass away, and then if you survive.” (A25) “A lot of people said I was courageous, people and heroic and words like that, but I never felt like that applied because I didn’t feel like I had a choice. I, I felt that this was just something that I had to go through to deal with, to survive.” (A26)
	Fear of recurrence and new cancers	“Anxious sometimes. […] especially if it’s a bad headache and I’m not sure why I get it, so like I don’t have sinus, I don’t have like a sinus problem that day if I have a headache, I’m like ok like you know I have […] because of my cancer, so like is. Is there a problem with my […].” (A14) “I don’t like the look of this or like I […] had a […] removed last year that I forced my family dr. to remove that she swore […] didn’t need to be removed. She was like “A15” it is not cancer. Like you’re […] years cancer free, it’s not cancer, definitely not.” (A15)
	Wish for autonomy	[*participant describes how one person told everyone their cancer diagnosis in a group discussion*] “And then of course everyone in the class was like, “oh my God, do you have cancer? When did you have cancer? Where was your cancer?” And […] everyone just wants to know all things then.” (A15) “A lot of things I found back then were talked about without me. I know I was young, but they, like, as I’ve grown up, I’m no longer close with my [*parent*]. So I was left to like research the pieces and kind of get the information back for myself, because I was excluded from a lot of like decisions and things impacting like my health and things down the road. So it would have been nice to have been included in more things regardless of how young I was or to have been provided with more of a summary of my care when I got a bit older.” (A20)
	Wish to leave cancer behind	“But some families still talk about it now and I’m like, well, that’s in the past, so, but definitely less as I’ve gotten older.” (A20) “I […] have a hard time even in the community, like I’ll run into people and they’re like, “oh my God, you were, you’re the little one that was sick that time”. Yeah, I was, but that was a long time ago.” (A15)

## Data Availability

Data that support the findings of this study are available from the researchers. However, restrictions apply to the availability of this data, and so data are not publicly available. The data used in this study cannot be made publicly available as patients were not consented to make their data publicly available or accessible. Transcripts and other data collected from the participants may be available upon reasonable request for researchers who meet the criteria for access to confidential data. Permission to obtain the data can be requested from Sevtap Savas (savas@mun.ca) and Research, Grant, and Contract Services (ris@mun.ca) at Memorial University of Newfoundland, St. John’s, NL, Canada, and the ethics approval shall be obtained from the Health Research Ethics Board (HREB), Ethics Office, Health Research Ethics Authority, Suite 200, 95 Bonaventure Avenue, St. John’s, NL, A1B 2X5, Canada.

## Supplementary Materials

The following supporting information can be downloaded at: https://www.mdpi.com/article/10.3390/curroncol33080494/s1, Supplementary Document S1: Interview guide [12,15,30,31,32,33]; Supplementary Document S2: Sociodemographic features of the participants [62].

## Abbreviations

The following abbreviations are used in this manuscript:

HREA	Health Research Ethics Authority
HREB	Health Research Ethics Board
ID	Identity Document
NL	Newfoundland and Labrador
PI	Principal Investigator
