# Supplementary material for: Exploring the Social and Stigma-Related Lived Experiences of Pediatric Cancer Survivors in a Canadian Province
Source: Curr Oncol. 2026 Aug 20;33(8):494. doi: 10.3390/curroncol33080494 (PMC13510854; doi:10.3390/curroncol33080494)
Supplement: Supplementary file 1 [file curroncol-33-00494-s001.zip › curroncol-4475645-supplementary.pdf]

Supplementary Document S1. Interview guide.

i. Ice-breaker questions

Ice-breaker Question 1: How are you today?

Prompts: How is your day going? Hope your day is going well.

Ice-breaker Question 2: Before we begin our chat today, do you have any questions for me or about the study?

Prompts: Did you have anything to clarify about the study? About this interview? About your rights as participants?

Ice-breaker Question 3: Can you tell me a bit about your experience of childhood cancer from what you can remember?

Prompts: How old were you? What stands out to you about your experience?

ii. Interview questions

Question 1) How were your life experiences since your diagnosis in terms of your relationships with others? Did you ever experience any relationship issues because of your cancer diagnosis (with friends, family members, peers at school, healthcare providers, and if relevant, in work environments)?

Prompts: How did your family members and friends interact with you after your diagnosis? Did you feel like your friends and family members changed in their approach to you after your cancer diagnosis? How did your doctor and teachers at school treat you and interact with you after your diagnosis? Did you work at all after your diagnosis? If so, how was your relationships with the co-workers and employers? Did their approach to you change because of cancer? Did their approach to you change over the years? Do you think you were treated well or differently after your cancer diagnosis?

Question 2) Did you receive support (financial, emotional, social, and so on) from others following your cancer diagnosis (in social, school, healthcare, and if relevant, work environments)?

Prompts: Do you think that you had adequate support after your cancer diagnosis? Did your family and friends support you? How about your doctor/nurse, your teachers at school and your coworkers if you have worked after your diagnosis? Did you feel supported or did you wish there were more support to you? Did the support levels change over the years?

Question 3) Do you think that you experienced stigma or discrimination because of cancer since your diagnosis (in social, school, healthcare, and if relevant, work environments)?

Lay definitions are as follows:

Stigma happens when we are negatively or unfairly treated by others because of our history of cancer. It may feel like being side-lined and left out because of cancer.

Discrimination happens when we have reduced or diminished opportunities because of our history of cancer. It may feel like you are not given what you normally deserve.

Prompts: Did you feel sidelined, marginalized or left out after your cancer diagnosis? Do you feel that you were stigmatized by others? Did you feel that others treated your negatively or unfairly because of your cancer history and made you feel uncomfortable? Did you or do you feel that you have had less opportunities in your personal life or career because of discrimination due to your cancer history? (e.g., when seeking social or romantic relationships, career opportunities, etc.). Did others make assumptions about you and your abilities and made you uncomfortable? Were there expectations from others that made you uncomfortable? For example, were you called “super hero” or “inspirational” for surviving pediatric cancer and did this make you feel guilty? If so, who were involved in these experiences? Your friends? Your family members? Your teachers at schools? Your healthcare provider? Your employer and coworkers? Did these experiences change over time?

Question 4) What were the effects of cancer-associated sidelining, stigma and discrimination on you and your well-being?

Prompts: Do you think that you were negatively affected by being sidelined, stigma or discrimination that happened because you had a history of cancer? How did you feel when you

were sidelined, stigmatized or discriminated against because of your cancer history? Did these experiences affect your mental health? Did you feel depressed or anxious because of these experiences? Did these experiences change over time?

Note: skip this question if the answer to Question 3 was no.

Question 5) How did you cope with cancer-associated stigma / discrimination and its consequences on you?

Prompts: How did you manage to address the effects of marginalization, unfair treatment, being stigmatized, being left out, or being subject to reduced or lost opportunities? Did you utilize coping mechanisms, such as talking to family, friends, healthcare providers, teachers, a trusted adult, or support group members? Did you practice mindfulness, exercise, or any other wellness related activity to deal with the emotions arisen because of the stigma or discrimination?

Note: skip this question if the answer to Question 3 was no.

Question 6) Did you wish, seek or receive clinical support, such as counselling and therapy, in order to cope with cancer associated stigma's effects?

Prompts: Did you need external support, such as from a counsellor, therapist, mental health providers, or a clinician in order to address the effects of cancer-associated stigma on your wellbeing and life?

Note: skip this question if the answer to Question 3 was no.

Wrap up

Thanking the participant and answering their final questions, if any.

References: [12, 15, 30-33].

Supplementary Documents S2. Sociodemographic features of the participants

Characteristics	N (%)
**Type of cancer**	Acute lymphoblastic leukemia; Pineal gland brain tumor; Osteosarcoma; Medulloblastoma; Rhabdomyosarcoma; Leukemia; Pineal germinoma
**Years passed since cancer diagnosis**	13 - 28 years (median: 20 years)
**Participant age range**	
10-18	1
19-25	1
26-32	3
33-39	2
**Marital status**	
Single	3
Legally Married	3
Common-law	1
Divorced/Separated	-
Widowed	-
Prefer not to Answer	-
Not applicable	-
**Sex at birth**	
Male	4
Female	3
Prefer not to Answer	-

**Gender**	
Male	4
Female	3
Non-binary	-
Other, please describe________________________	-
Prefer not to Answer	-
**Highest level of education**	
Less than high school	-
High school Diploma	3
Trade or College Diploma	2
University, undergraduate degree	-
University, graduate degree	2
Prefer not to Answer	-
Not applicable	-
***Rural or urban location**	
I live in an urban city with a population of 5,000 or more. These include St. John's, Bay Roberts, Corner Brook, Gander, Grand Falls-Windsor, Clarenville, Deer Lake, Happy Valley-Goose Bay, Labrador City, Marystown, or Stephenville	6
I live in a rural area/town that is not listed above	1
Prefer not to Answer	-
**Health authority region/zone**	
Eastern	6
Central	1
Western	-
Labrador Grenfell	-

Prefer not to Answer	-
**Race/ethnicity**	
Indigenous (First Nations, Inuit, Metis)	-
Latin-American (e.g. Argentinian, Chilean, Cuban)	-
Asian – East (e.g. Chinese, Japanese)	-
Asian – South (e.g. Indian, Sri Lankan, Indo-Caribbean)	-
Asian – South East (e.g. Vietnamese, Filipino)	-
Middle Eastern (e.g. Egyptian, Iranian, Israeli, Palestinian)	-
White/European (e.g. English, Greek, Italian, Serbian)	7 (100%)
Black – Africa (e.g. Ghanaian, Somalian)	-
Black – North American	-
Black – Caribbean Region (e.g. Jamaican, Trinidadian, Barbadian)	-
Mixed heritage (Please specify) ___________________	-
Other(s), please specify ______________________	-
Prefer not to Answer	-
**Annual household income**	
Under $20,000	1
$ 20,000 - $ 39,999	-
$ 40,000 - $ 59,999	-
$ 60,000 - $ 74,999	1
$ 75,000 - $ 99,999	2
$ 100,000 - $ 149,999	2
$150,000 or more	1
Prefer not to Answer	-

Not applicable	-
**Work after cancer diagnosis**	
Yes	6
No	-
Prefer not to Answer	-
Not applicable	1
**Self-employed at anytime after diagnosis**	
Yes	2
No	4
Sometimes	-
Prefer not to Answer	-
Not applicable	- (no response=1)

Percentages do not add up to 100 because of rounding. N: total number. Urban cities include St. John's, Bay Roberts, Corner Brook, Gander, Grand Falls-Windsor, Clarenville, Deer Lake, Happy Valley-Goose Bay, Labrador City, Marystown, or Stephenville - based on the definition by the Newfoundland and Labrador Statistics Agency [62].
